# AI Integration in Brazilian Master’s Curricula and Its Implications in Dental Education

**DOI:** 10.1155/ijod/9974810

**Published:** 2026-08-07

**Authors:** Jessica Nobrega Dantas, Marco Antonio Dias da Silva

**Affiliations:** ^1^ Academic Unity of Dentistry, Federal University of Campina Grande, Av. Universitária, s/n – Santa Cecilia, Patos, PB, 58708-110, Brazil, ufcg.edu.br; ^2^ Research Unit of Population Health, Faculty of Medicine, University of Oulu, P.O. Box 5000, FI-90014, Oulu, Finland, oulu.fi; ^3^ Department of Clinical and Experimental Sciences, University of Brescia, Brescia, Italy, unibs.it

**Keywords:** artificial intelligence, curriculum reform, educational technology, postgraduate education, teacher training

## Abstract

The integration of artificial intelligence (AI) into dental education is an issue capable of empowering professionals and revolutionizing patient outcomes. Nevertheless, there is a lack of data regarding the depth of AI adoption within Brazilian Dental Master’s Programs. This research aimed to assess the availability of AI‐related content in Brazilian Master’s Programs. A total of 92 master’s programs were analyzed using the governmental Sucupira database. The assessment considered the official program quality ratings, geographic regions, the type of master’s, and the administrative category. Within the 2161 courses examined, AI‐related content was identified in only 13 programs. An association was observed between the best programs and the inclusion of AI into the curriculum (*p* < 0.001). Conversely, the type of master’s degree, geographic location, or the administrative category presented no correlation (*p* > 0.05). It was concluded that the integration of AI within Brazilian Dental Master’s Programs remains restricted to specific courses. These results potentially reflect a scarcity of AI specialists and the necessity for curricular reforms emphasizing the role of the master’s programs in training the future dental educators.

## 1. Introduction

Artificial intelligence (AI) is improving facial recognition, translation, and virtual assistants, promoting more personalized solutions [1–6]. AI evolution depends on the database’s quality and capacity to adapt to different devices [3]. In dentistry, AI helps develop intelligent systems that can perform some tasks better than humans, allowing professionals to spot hidden patterns [7]. Nowadays, AI‐powered tools have changed diagnosis [8–13], prognosis predictions [7–11, 14], and drug discovery [15], benefiting dental professionals and leading to better patient outcomes [16, 17].

Effective AI integration in dental education is crucial but requires moving beyond symbolic use toward meaningful pedagogy. This approach fosters critical thinking about AI’s limits and ethical implications to prevent misuse [18]. While AI offers treatment improvements [19, 20], dentists must address accountability, transparency, and algorithmic bias [21]. It has been shown that students often accept AI for administrative support but remain cautious about its use in assessment [22]. Therefore, pedagogy integration must balance technical competency with the “human” skills, such as empathy, that AI cannot replace.

Interest in AI in dentistry is increasing as it transforms education and clinical practice [23]. Despite this, students have limited understanding and concerns about AI [24]. However, the formal integration of novelties into the dental curriculum usually faces significant structural lags. Linking innovation with formal education is a challenge; this complex transition requires time to merge ongoing methods with emerging technologies. While integrating AI depends on well‐trained teachers, limited resources and faculty availability may rise as major restrictions [25].

How extensively AI concepts have been incorporated into dental education in Brazil remains unclear. In Brazil’s higher educational system, PhDs are research‐oriented, whereas master’s prepare dental teachers. To address this knowledge gap, the present study investigated how AI concepts are being integrated into Brazilian Dental Master’s Programs’ curricula, specifically focusing on the programs responsible for training the next generation of dental educators.

## 2. Material and Methods

This quantitative documental research investigated AI content in Brazilian Dental Master’s Programs. To ensure a scientific baseline, this research focused on structural integration. We prioritized the AI concepts present in official institutional documents within the government database, excluding informal or extracurricular instruction. In accordance with the Brazilian regulations, as the research utilizes only anonymized, publicly accessible institutional information and does not involve human subjects, this study is exempt from ethical clearance.

### 2.1. Data Source and Sampling

Between April and June 2023, all Brazilian Master’s programs in dentistry were examined. To guarantee standardization, reproducibility, accuracy, and completeness, the official list of programs was obtained from the Governmental database “https://sucupira.capes.gov.br/sucupira”. All websites were accessed through the link provided by the government platform.

Since, in Brazil, master’s programs are obliged to maintain updated official information, absent or broken links resulted in the exclusion of the program. This criterion was established to prevent the inclusion of nonofficial or outdated information, maintaining the integrity of the data source.

### 2.2. Website and Syllabus Content Evaluation

The official website of each master’s program was accessed and reviewed. Data extraction included information about courses and their descriptions or program syllabi. The syllabi and course list were searched by using the terms “artificial intelligence,” “machine learning,” and “AI” in Portuguese and English. The terms “robot” and “robotic” were also included due to their relationship with AI‐driven protocols in the current dental practice. A strict exclusion criterion was applied to distinguish between general digital technologies and AI. Digital technologies where AI is not the primary component, such as “CAD/CAM,” “digital radiography,” and “intraoral scanning,” were excluded. All the keywords found were only considered positive for AI integration when the associated syllabus explicitly detailed AI‐driven protocols, ensuring no false inclusions.

### 2.3. Data Collection, Enrichment, Correlations, and Statistical Analysis

The study incorporated the official program quality score (CAPES), geographic region, and curricular data into the dataset. We addressed the theoretical framework qualitatively categorizing AI use into functional integration (technical skills, diagnostic tools, and clinical software applications) or pedagogical integration (bioethics, societal impact, and critical reasoning).

Data were submitted for statistical analysis using Jamovi (v.2.3.21). Following the descriptive analysis, potential associations regarding the incorporation of AI in dental programs were explored under four distinct criteria: (1) program quality rating, (2) geographic region, (3) master’s degree type (professional or academic), and (4) the administrative category (public or private). Chi‐squared or Fisher’s exact test was used, respecting the sample size of each analysis.

Grammarly was employed for grammatical and readability refinement. LLMs and NotebookLM were used for language editing and to assist in the synthesis of literature. Reference management and citation formatting were conducted using Mendeley. These tools were used for linguistic and technical support. We maintain full accountability for the integrity of the work.

## 3. Results

### 3.1. Data Source, Website, and Syllabus Content Evaluation

The study examined 92 Brazilian Dental Master’s Programs, comprising 75 academic and 17 professional programs. During the assessment of the government platform, seven websites (7.6%) were inaccessible; consequently, 85 programs, 2161 disciplines, and 1523 course syllabi or course descriptions were analyzed. The comprehensive assessment also included data on infrastructure and research areas (Table 1).

**Table 1:** Names of courses and research areas positive for the keywords “artificial intelligence,” “machine learning,” “robot,” “robotics,” and “AI.”

Description of	Subject
1 Course	Methods of Analysis Applied to Tomographic Images
1 Course	Digital Dentistry and Basis of Artificial Intelligence
1 Course	Current Aspects in Research in Digital Dentistry and Artificial Intelligence
1 Course	Teledentistry, Telehealth, and Digital Health: New Forms of Care and Teaching after the COVID‐19 Pandemic
1 Course	Innovation, Technology, and Entrepreneurship in Dental Materials and Oral Rehabilitation
7 Courses	Ethics, Bioethics, and Biological Diversity of Interest in Dentistry
1 Research area	Teaching in Dentistry

### 3.2. AI Content Identification

AI‐related content was identified in only 0.55% of the 2161 disciplines analyzed. Based on the syllabus descriptions (Table 1), two patterns were observed: functional integration, focusing on digital imaging, innovation, and rehabilitation (five courses); and the most frequent pedagogical integration, concentrated in ethics and teaching methodology (seven courses and one research area). Interestingly, most occurrences were identified within the disciplines of ethics and bioethics.

### 3.3. Data Collection and Statistical Analysis

AI content was found in 12 programs in the Southeast and one in the South (Table 2), regions that host most programs and the best‐rated institutions in the country. All programs identified were in public institutions.

**Table 2:** List of Brazilian Dental Master’s Programs where keywords related to AI were found, categorized by institution, program type, administrative category, geographic region, and CAPES evaluation.

Program	Type	Admin	Region	Score
Applied Dental Sciences	Academic	Public	Southeast	6
Dentistry	Academic	Public	Southeast	5
Oral Diagnosis	Academic	Public	Southeast	4
Dental Sciences	Academic	Public	Southeast	5
Oral Rehabilitation	Academic	Public	Southeast	5
Dental Biology	Academic	Public	Southeast	5
Dentistry	Academic	Public	Southeast	7
Dental Materials	Academic	Public	Southeast	6
Collective Health Dentistry	Professional	Public	Southeast	5
Clinical Dentistry	Academic	Public	Southeast	7
Oral Medicine and Pathology	Academic	Public	Southeast	6
Oral Radiology	Academic	Public	Southeast	5
Dentistry	Academic	Public	South	6

Statistical analysis of the incorporation of AI concepts in dental programs based on four distinct criteria demonstrated:

1. No correlation between the presence of AI‐related content and the type of master’s degree (professional or academic) (*p*  > 0.05).
2. No correlation between the presence of AI‐related content and the geographic region of the program (*p*  > 0.05).
3. No correlation between the administrative category of the program (public or private) and the offering of AI‐related content (*p*  > 0.05).
4. Positive association between higher CAPES ratings and the presence of AI content (Grade 7: 66% and Grade 6: 50%) (*p*  < 0.001) (Table 3). In opposition, AI‐related content could not be found in most programs with a CAPES score of 5 or less (*p*  > 0.05).

**Table 3:** Distribution of Brazilian Dental Master’s Programs offering AI‐related content according to their CAPES score.

Score	Number of programs	% Observed
7	2	66.7 ^∗^
6	4	50.0 ^∗^
5	6	23.1
4	1	2.8
3	0	0

^∗^ *p* < 0.001.

## 4. Discussion

AI integration into health education has become a priority [18, 25], with current studies debating the essential competencies required for future professionals. In nursing, the use of AI‐chatbots [26] and the incorporation of gen‐AI into the undergraduate curricula are growing trends that confirm AI’s disruptive potential [27]. In medicine, similarly, AI has been addressed as a tool for training medical residents [28–30] in complex tasks.

In dentistry, however, the successful AI assimilation in education requires dealing with real‐life obstacles [22], and dental education still struggles to move from theoretical awareness to practical integration. Educators are trying to understand students’ perceptions and the ways students are already utilizing AI in different dental specialties [24]. It is known that AI is capable of surpassing traditional diagnostic methods in identifying cavities, dental plaque, gingivitis, and alveolar bone loss [12, 15, 31] while enhancing efficiency and safety [32].

Despite all that, the discussion regarding curricular reform is progressing in small steps [21, 25, 33]; in Brazil, it looks even more discrete [24]. The present study exposes a significant structural lag, as most programs have not yet formally integrated AI into their syllabi.

The present research raises an alert regarding the implications of delaying the integration of AI concepts in dental education. Worldwide, many dental educators are excellent clinicians who often identify themselves more strongly with their clinical and research roles than with their identity as educators [34–36]. Consequently, they tend to replicate the teaching models, methods, and concepts they experienced as students [36].

This is perfectly understandable since formal pedagogical training is not part of the health course curriculum, shaping dental educators’ identity [37]. In contrast, when early‐career educators receive formal training, they are able to transcend instinctive instruction and develop a science‐based learning, which allows them to adapt to and innovate more easily [36, 38]. Therefore, if future dental educators are not exposed to AI concepts during their master’s training, their readiness and keenness to introduce further curricular changes will remain quite limited.

### 4.1. Factors Influencing AI Integration

Although only 13 programs offered AI content, some institutions already emphasize AI fundamentals as part of an innovative and technological approach. Our findings demonstrate that in these curricula, AI applications range from basic sciences to clinical analysis. These courses aim to provide master’s students with the skills to use AI in clinical practice and research, characterizing a functional integration of AI 28 focused on the improvement of dental services [39].

The use of AI for image analysis and treatment planning and diagnosis [12, 13, 16, 39] has influenced research within these programs as well. A course titled “Current Aspects in Research in Digital Dentistry and Artificial Intelligence” exemplifies AI integration and the importance of interdisciplinary collaborations. Additionally, AI in bioethics courses represents a pedagogical integration of AI and the commitment to graduating better‐prepared professionals (Table 1). Interestingly, the presence of AI content does not correlate with the type of master’s degree, its administrative category, or the geographical region of universities.

The scarcity of AI specialists within dental schools is a challenge. Most educators are dedicated to specific clinical subjects, and assessing students’ needs is necessary to propose curricular reforms [40]. Educational leadership and resource availability are considered major factors for integrating AI into curricula [25, 41, 42]. Institutions that recognize the transformative potential of AI and prioritize curriculum development are more likely to incorporate AI concepts [25].

Courses covering AI basics, machine learning, and deep learning show diverse ways of inserting AI in Brazilian Dental Master’s Programs. It is essential to understand that AI principles are not exclusive to mathematics, engineering, and computer science students [25]. While more resourceful institutions may find it easier to invest in AI courses, it is also important to consider the importance of hiring faculty able to incorporate AI‐related content into dental education [21, 25, 42, 43].

### 4.2. Program Quality Score and the Offering of AI Content

This study found that programs scoring 7 or 6 were more likely (*p*  < 0.01) to include AI‐related content (Table 3) in their curricula. This suggests that research‐intensive institutions may be better positioned to officially adopt technological innovations due to better‐structured programs, strong faculty, and a research‐oriented culture [44].

For institutions with more limited resources, AI adoption may remain at the level of symbolic inclusion with no full functional or pedagogical integration into the coursework [21, 31].

Corroborating our findings, all universities offering AI content (Table 2) are listed among the top‐ranked dental schools in Brazil [45, 46]. These data suggest that quality assurance agencies should support curriculum enhancement, focusing not only on students but also on the qualifications of the faculty and staff for an effective digital transition.

### 4.3. Ethical Considerations and Data Quality

As AI becomes integrated into practice, ethical considerations regarding patient privacy, data security, and algorithmic bias must be addressed. Dental professionals need skills to navigate these ethical challenges responsibly [18, 21, 41, 42, 47]. Moreover, ensuring the quality of data used in AI applications is crucial as inaccurate or biased data can lead to incorrect diagnoses and treatments [47]. Therefore, dental programs should emphasize the importance of data quality and include training in understanding data collection, curation, and validation.

### 4.4. Preparing Students for the Future

The implications of AI integration in dental education are significant. AI provides dental professionals with real‐time patient data, which can enhance treatment personalization [8, 10–12, 14, 32, 48, 49]. Students familiar with AI will be better positioned to improve patient care [39, 42]. However, while AI supports clinical decision‐making, it must not replace the clinical judgment of the professional or student [42].

The main challenge in dental education is ensuring that students are taught to use AI ethically and responsibly, understanding the limits and the risks in future professional settings [22]. By addressing these needs, dental schools can develop curricula that prepare students for future challenges and opportunities [21, 22, 41].

### 4.5. Limitations

In this study, seven websites (7.6%) could not be accessed. However, to ensure reproducibility, only the official government database was used. In addition, only quantitative data were extracted from websites, and no qualitative data were used. In this study, only explicit curricular intent was measured through keyword identification in course titles and descriptions. The impact of institutional culture, faculty expertise, and funding may have played significant roles in determining the AI content in programs. This approach may not have captured the pedagogical depth, actual workload, or specific learning outcomes provided in the courses. We cannot omit, as well, that concepts may be taught by innovative educators who introduce novelties informally into the syllabus.

Future research should address these qualitative aspects to further enhance our understanding of AI’s role in dental education.

Dentistry is still adapting to AI, and it is still complex [42] in both clinical and educational settings. This study was not designed to evaluate the quality of the instruction or the level of technical expertise with which these concepts are delivered.

## 5. Conclusion

The integration of AI‐related content into dental master’s programs in Brazil is limited and associated with specific courses. It may suggest an alignment between research intensity and the adoption of innovative methods. However, the lack of AI specialists and the lag in curricular reform remain as challenges. The finding emphasizes the need for changes to ensure the effective integration of AI in Brazilian Master’s Programs, since one of their main roles is training future dental educators.

## Author Contributions

**Jessica Nobrega Dantas**: methodology, software, data curation, investigation, writing – original draft. **Marco Antonio Dias da Silva**: conceptualization, methodology, formal analysis, supervision, validation, visualization, writing – review and editing, funding acquisition.

## Funding

The research leading to this publication was cofunded by the European Union’s Horizon Europe Research and Innovation under the Marie Skłodowska‐Curie Actions grant agreement 2430429511, grant agreement number 101126602 (Data4Healthcare), and the University of Oulu.

## Disclosure

The preprint version of this article has already been published in the ResearchSquare [50].

## Conflicts of Interest

The authors declare no conflicts of interest.

## Data Availability

The corresponding author can provide the data of this study by responding to a scientific and reasonable request. The raw and updated data are available in the Sucupira database.

## References

[1] Lugano G. , Virtual Assistants and Self-Driving Cars, *Proceedings of 2017 15th International Conference on ITS Telecommunications (ITST 2017)*, 2017, Warsaw, Poland, Institute of Electrical and Electronics Engineers (IEEE), Published online July 7, 2017 10.1109/ITST.2017.7972192.

[2] Feng S. , Yan X. , Sun H. , Feng Y. , and Liu H. X. , Intelligent Driving Intelligence Test for Autonomous Vehicles With Naturalistic and Adversarial Environment, Nature Communications. (2021) 12, no. 1, 10.1038/s41467-021-21007-8, 748. PMC7854639 33531506

[3] Apell P. and Eriksson H. , Artificial Intelligence (AI) Healthcare Technology Innovations: The Current State and Challenges From a Life Science Industry Perspective, Technology Analysis and Strategic Management. (2023) 35, no. 2, 179–193, 10.1080/09537325.2021.1971188.

[4] Reda A. and Aoued B. , Artificial Neural Network-Based Face Recognition, *International Symposium on Control, Communications and Signal Processing, ISCCSP*, 2004, Hammamet, Tunisia, Institute of Electrical and Electronics Engineers (IEEE), 439–442, Published Online 10.1109/ISCCSP.2004.1296323.

[5] Kondratyeva M. N. and Svirina D. D. , AIP Conference Proceedings, 10th Asian International Conference on Fluid Machinery. (2022) 2467, no. 1, 10.1063/5.0093020, 040011.

[6] Steck H. , Baltrunas L. , Elahi E. , Liang D. , Raimond Y. , and Basilico J. , Deep Learning for Recommender Systems: A Netflix Case Study, AI Magazine. (2021) 42, no. 3, 7–18, 10.1609/aimag.v42i3.18140.

[7] Jiang F. , Jiang Y. , and Zhi H. , et al.Artificial Intelligence in Healthcare: Past, Present and Future, Stroke and Vascular Neurology. (2017) 2, no. 4, 230–243, 10.1136/svn-2017-000101. 29507784 PMC5829945

[8] Engels P. , Meyer O. , and Schönewolf J. , et al.Automated Detection of Posterior Restorations in Permanent Teeth Using Artificial Intelligence on Intraoral Photographs, Journal of Dentistry. (2022) 121, 10.1016/j.jdent.2022.104124, 104124. 35395346

[9] Lee S. J. , Chung D. , and Asano A. , et al.Diagnosis of Tooth Prognosis Using Artificial Intelligence, Diagnostics. (2022) 12, no. 6, 10.3390/diagnostics12061422, 1422. 35741232 PMC9221626

[10] Mine Y. , Iwamoto Y. , and Okazaki S. , et al.Detecting the Presence of Supernumerary Teeth During the Early Mixed Dentition Stage Using Deep Learning Algorithms: A Pilot Study, International Journal of Paediatric Dentistry. (2022) 32, no. 5, 678–685, 10.1111/ipd.12946. 34904304

[11] Keser G. , Bayrakdar İ. Ş. , Pekiner F. N. , Çelik Ö. , and Orhan K. , A Deep Learning Algorithm for Classification of Oral Lichen Planus Lesions From Photographic Images: A Retrospective Study, Journal of Stomatology, Oral and Maxillofacial Surgery. (2023) 124, no. 1, 10.1016/j.jormas.2022.08.007, 101264. 35964938

[12] Lee J.-H. , Kim D.-H. , Jeong S.-N. , and Choi S.-H. , Detection and Diagnosis of Dental Caries Using a Deep Learning-Based Convolutional Neural Network Algorithm, Journal of Dentistry. (2018) 77, 106–111, 10.1016/j.jdent.2018.07.015. 30056118

[13] Zhu J. , Chen Z. , and Zhao J. , et al.Artificial Intelligence in the Diagnosis of Dental Diseases on Panoramic Radiographs: A Preliminary Study, BMC Oral Health. (2023) 23, no. 1, 10.1186/s12903-023-03027-6, 358. 37270488 PMC10239110

[14] Chau R. C. W. , Chong M. , and Thu K. M. , et al.Artificial Intelligence-Designed Single Molar Dental Prostheses: A Protocol of Prospective Experimental Study, PLoS ONE. (2022) 17, no. 6, 10.1371/journal.pone.0268535, e0268535. 35653388 PMC9162350

[15] Lin Y. , Zhang Y. , Wang D. , Yang B. , and Shen Y.-Q. , Computer Especially AI-Assisted Drug Virtual Screening and Design in Traditional Chinese Medicine, Phytomedicine. (2022) 107, 10.1016/j.phymed.2022.154481, 154481. 36215788

[16] Tam W. , Huynh T. , Tang A. , Luong S. , Khatri Y. , and Zhou W. , Nursing Education in the Age of Artificial Intelligence Powered Chatbots (AI-Chatbots): Are We Ready yet?, Nurse Education Today. (2023) 129, 10.1016/j.nedt.2023.105917, 105917. 37506622

[17] Laurenza E. , Quintano M. , Schiavone F. , and Vrontis D. , The Effect of Digital Technologies Adoption in Healthcare Industry: A Case Based Analysis, Business Process Management Journal. (2018) 24, no. 5, 1124–1144, 10.1108/BPMJ-04-2017-0084.

[18] Thurzo A. , Strunga M. , Urban R. , Surovková J. , and Afrashtehfar K. I. , Impact of Artificial Intelligence on Dental Education: A Review and Guide for Curriculum Update, Education Sciences. (2023) 13, no. 2, 10.3390/educsci13020150, 150.

[19] Bichu Y. M. , Hansa I. , Bichu A. Y. , Premjani P. , Flores-Mir C. , and Vaid N. R. , Applications of Artificial Intelligence and Machine Learning in Orthodontics: A Scoping Review, Progress in Orthodontics. (2021) 22, no. 1, 10.1186/s40510-021-00361-9, 18. 34219198 PMC8255249

[20] Alowais S. A. , Alghamdi S. S. , and Alsuhebany N. , et al.Revolutionizing Healthcare: The Role of Artificial Intelligence in Clinical Practice, BMC Medical Education. (2023) 23, no. 1, 10.1186/s12909-023-04698-z, 689. 37740191 PMC10517477

[21] Davenport T. and Kalakota R. , The Potential for Artificial Intelligence in Healthcare, Future Healthcare Journal. (2019) 6, no. 2, 94–98, 10.7861/futurehosp.6-2-94. PMC6616181 31363513

[22] Kavadella A. , Dias da Silva M. A. , Kaklamanos E. G. , Stamatopoulos V. , and Giannakopoulos K. , Evaluation of ChatGPT’s Real-Life Implementation in Undergraduate Dental Education: Mixed Methods Study, JMIR Medical Education. (2024) 10, no. 1, 10.2196/51344, e51344. 38111256 PMC10867750

[23] Shimizu I. , Kasai H. , and Shikino K. , et al.Developing Medical Education Curriculum Reform Strategies to Address the Impact of Generative AI: Qualitative Study, JMIR Medical Education. (2023) 9, no. 1, 10.2196/53466, e53466. 38032695 PMC10722362

[24] Pauwels R. and Del Rey Y. C. , Attitude of Brazilian Dentists and Dental Students regarding the Future Role of Artificial Intelligence in Oral Radiology: A Multicenter Survey, Dentomaxillofacial Radiology. (2021) 50, no. 5, 10.1259/dmfr.20200461, 20200461. 33353376 PMC8231677

[25] Castonguay A. , Farthing P. , and Davies S. , et al.Revolutionizing Nursing Education through Ai Integration: A Reflection on the Disruptive Impact of ChatGPT, Nurse Education Today. (2023) 129, 10.1016/j.nedt.2023.105916, 105916. 37515957

[26] Lindqwister A. L. , Hassanpour S. , Lewis P. J. , and Sin J. M. , AI-RADS: An Artificial Intelligence Curriculum for Residents, Academic Radiology. (2021) 28, no. 12, 1810–1816, 10.1016/j.acra.2020.09.017. 33071185 PMC7563580

[27] Ng K. H. and Wong J. H. D. , A Clarion Call to Introduce Artificial Intelligence (AI) in Postgraduate Medical Physics Curriculum, Physical and Engineering Sciences in Medicine. (2022) 45, no. 1, 1–2, 10.1007/s13246-022-01099-2. 35006576

[28] Aljulayfi I. S. , Almatrafi A. H. , and Althubaitiy R. O. , et al.The Potential of Artificial Intelligence in Prosthodontics: A Comprehensive Review, Medical Science Monitor. (2024) 30, 10.12659/MSM.944310, 944310. PMC11178143 38840416

[29] Revilla-León M. , Gómez-Polo M. , and Barmak A. B. , et al.Artificial Intelligence Models for Diagnosing Gingivitis and Periodontal Disease: A Systematic Review, The Journal of Prosthetic Dentistry. (2023) 130, no. 6, 816–824, 10.1016/j.prosdent.2022.01.026. 35300850

[30] Putra R. H. , Doi C. , Yoda N. , Astuti E. R. , and Sasaki K. , Current Applications and Development of Artificial Intelligence for Digital Dental Radiography, Dentomaxillofacial Radiology. (2022) 51, no. 1, 10.1259/dmfr.20210197, 20210197. 34233515 PMC8693331

[31] Thurzo A. , Urbanová W. , and Novák B. , et al.Where Is the Artificial Intelligence Applied in Dentistry? Systematic Review and Literature Analysis, Healthcare. (2022) 10, no. 7, 10.3390/HEALTHCARE10071269, 1269. 35885796 PMC9320442

[32] Islam N. M. , Laughter L. , and Sadid-Zadeh R. , et al.Adopting Artificial Intelligence in Dental Education: A Model for Academic Leadership and Innovation, Journal of Dental Education. (2022) 86, no. 11, 1545–1551, 10.1002/JDD.13010. 35781809

[33] Ceraulo S. , Caccianiga P. , Casto C. , Ceraulo I. , and Caccianiga G. , Dental Prosthetic Rehabilitation Interventions in Elderly Patients Hospitalized in the Nursing Homes of the Lombardy Region: A Retrospective Study, Healthcare. (2022) 10, no. 11, 10.3390/healthcare10112328, 2328. 36421652 PMC9690150

[34] Stone S. , Ellers B. , Holmes D. , Orgren R. , Qualters D. , and Thompson J. , Identifying Oneself as a Teacher: The Perceptions of Preceptors, Medical Education. (2002) 36, no. 2, 180–185, 10.1046/j.1365-2923.2002.01064.x. 11869447

[35] Barber J. R. G. , Park S. E. , and Jensen K. , et al.Facilitators and Barriers to Teaching Undergraduate Medical Students in General Practice, Medical Education. (2019) 53, no. 8, 778–787, 10.1111/medu.13882. 31012131

[36] Hervas G. and Medina J. L. , Navigating Without a Map: Unpacking Pedagogical Viewpoints of Health Sciences Educators Without Educational Training, Medical Science Educator. (2025) 35, no. 6, 3115–3122, 10.1007/s40670-025-02528-z. 41798388 PMC12961023

[37] Snook A. G. , Schram A. B. , Jones B. D. , and Sveinsson T. , Factors Predicting Identity as Educators and Openness to Improve: An Exploratory Study, Medical Education. (2019) 53, no. 8, 788–798, 10.1111/medu.13909. 31131926

[38] Davey R. , Da Silva A. L. S. , and Bezdickova M. , Pedagogy in Practice: A Qualitative Analysis of Evidence-Based Teaching Methods Used by Graduate-Entry Near-Peer Medical Educators, Frontiers in Medicine. (2026) 13, 10.3389/fmed.2026.1757648, 1757648. 41836923 PMC12982050

[39] Schwendicke F. , Samek W. , and Krois J. , Artificial Intelligence in Dentistry: Chances and Challenges, Journal of Dental Research. (2020) 99, no. 7, 769–774, 10.1177/0022034520915714. 32315260 PMC7309354

[40] Kavadella A. , Uribe S. E. , and da Silva M. A. D. , et al.Dental Students’ Knowledge, Perceptions, and Educational Needs Regarding Artificial Intelligence: A Multinational Cross-Sectional Survey, BMC Medical Education. (2026) 26, no. 1, 10.1186/s12909-026-08699-6, 8699. PMC12977397 41652399

[41] Kim C. S. , Samaniego C. S. , Sousa Melo S. L. , Brachvogel W. A. , Baskaran K. , and Rulli D. , Artificial Intelligence (A.I.) in Dental Curricula: Ethics and Responsible Integration, Journal of Dental Education. (2023) 87, no. 11, 1570–1573, 10.1002/jdd.13337. 37489621

[42] Johnson K. B. , Wei W. Q. , and Weeraratne D. , et al.Precision Medicine, AI, and the Future of Personalized Health Care, Clinical and Translational Science. (2021) 14, no. 1, 86–93, 10.1111/cts.12884. 32961010 PMC7877825

[43] Rodway P. and Schepman A. , The Impact of Adopting AI Educational Technologies on Projected Course Satisfaction in University Students, Computers and Education: Artificial Intelligence. (2023) 5, 10.1016/j.caeai.2023.100150, 100150.

[44] Instituto de Pesquisas Jardim Botânico do Rio de Janeiro , Graduate Programs Progress in the CAPES Evaluation, 2026, Instituto de Pesquisas Jardim Botânico do Rio de Janeiro (JBRJ), Rio de Janeiro, Brazil, Accessed January 20, 2026 https://www.gov.br/jbrj/en/subjects/news/graduate-programs-progress-in-the-capes-evaluation.

[45] Xuanxiao , QS World University Rankings for Dentistry 2025 | Top Universities, Accessed 13 May, 2026 https://xuanxiao.org/en/rankings/qs/subject/dentistry/2025.

[46] Times Higher Education (THE) , World University Rankings 2026, 2026, Times Higher Education (THE), London, United Kingdom, Accessed 13 May, 2026 https://www.timeshighereducation.com/world-university-rankings/latest/world-ranking.

[47] Rasteau S. , Ernenwein D. , Savoldelli C. , and Bouletreau P. , Artificial Intelligence for Oral and Maxillo-Facial Surgery: A Narrative Review, Journal of Stomatology, Oral and Maxillofacial Surgery. (2022) 123, no. 3, 276–282, 10.1016/j.jormas.2022.01.010. 35091121

[48] Agrawal P. and Nikhade P. , Artificial Intelligence in Dentistry: Past, Present, and Future, Cureus. (2022) 14, no. 7, 10.7759/cureus.27405, e27405. 36046326 PMC9418762

[49] Sakai T. , Li H. , and Shimada T. , et al.Development of Artificial Intelligence Model for Supporting Implant Drilling Protocol Decision Making, Journal of Prosthodontic Research. (2023) 67, no. 3, 360–365, 10.2186/jpr.JPR_D_22_00053. 36002334

[50] Dantas J. N. and Dda Silva M. A. , Decoding AI Curricular Integration: Navigating Course Quality in Brazilian Dental Master’s Programs, 2023, 6, Published Online October 10.21203/rs.3.rs-3395718/v1.

